# Nestedness patterns and the role of morphodynamics and spatial distance on sandy beach fauna: ecological hypotheses and conservation strategies

**DOI:** 10.1038/s41598-018-22158-3

**Published:** 2018-02-28

**Authors:** Helio H. Checon, Guilherme N. Corte, Yasmina M. L. Shah Esmaeili, A. Cecilia Z. Amaral

**Affiliations:** 1 Departamento de Biologia Animal, Instituto de Biologia, Universidade Estadual de Campinas, Monteiro Lobato St., 255, CEP 13083-862 Campinas, São Paulo Brazil; 2 Instituto Oceanográfico, Universidade de São Paulo (USP), Praça do Oceanograifco, 191, CEP 05508-120, São Paulo, SP Brazil; 3 Programa de Pós-Graduação em Ecologia, Departamento de Biologia Animal, Instituto de Biologia, Universidade Estadual de Campinas, Monteiro Lobato St., 255, CEP 13083-862 Campinas, São Paulo, SP Brazil

## Abstract

Sandy beach fauna is hypothesized to be mainly structured by environmental variables. As such, it is expected that morphodynamic characteristics are limiting factors, and the species pool inhabiting harsher reflective beaches would be a subset of (i.e., nested in) the fauna of nearby dissipative beaches. We investigated the existence of a nestedness pattern in sandy beach assemblages, as well as the contribution of environmental and spatial variables (i.e., factors that potentially affect an assemblage regardless of environmental conditions - typically related to distance between sites and dispersal of organisms) on sandy beach macrobenthic fauna. Dissipative beaches had higher species richness than reflective beaches but we found no nestedness pattern. Furthermore, almost every beach showed exclusive species. Spatial variables exerted stronger influence on macrobenthic assemblages than local environmental variables. Our results therefore suggest that local and small-scale recruitment is the predominant process structuring macrobenthic assemblages. These results bring important implications for sandy beach conservation: given that spatial distance is an important factor structuring macrobenthic fauna and different sandy beaches harbor different pools of species, conservation programs need to focus on sandy beaches across large spatial scales and with varied morphodynamic characteristics in order to preserve coastal biodiversity.

## Introduction

Sandy beaches dominate the ocean shores of temperate and tropical coastlines^1,2^ and provide habitats for a well-adapted and diversified fauna, as well as key socioeconomic goods and services such as shoreline protection, nutrient cycling and fisheries^3^. Sandy beaches therefore play an important role in biodiversity conservation and in sustaining human populations. Despite their importance from both an environmental and social point of view, sandy beaches are still understudied when compared to other coastal systems^4–6^.

Over the past decades, however, a significant amount of research has been conducted and a variety of hypotheses concerning sandy beach ecosystems and communities have been developed. It is currently well known that sandy beaches are strongly influenced by environmental variables such as wave action, tides and sediment type^7,8^, and that the interaction of these variables determine a morphodynamic continuum from dissipative (beaches with gentle slopes, fine sands, wide surf zones and low hydrodynamic stress) to reflective beaches (with steep slopes, coarser grains, narrow surf zones and high hydrodynamic stress)^9–11^. It is also known that morphodynamic characteristics are key variables structuring sandy beach fauna^1^.

As a consequence of these determining environmental factors, the main hypotheses explaining the distribution patterns of intertidal macrofaunal organisms in sandy beaches at the community level (e.g., Swash Exclusion Hypothesis (SEH)^12^ and Multicausal Environmental Severity Hypothesis (MESH)^13^) are related to the Auto-Ecological Hypothesis^14^, which states that biological communities are structured by independent responses of individual species to the physical environment (i.e., species occurrence depends mainly on the individual capability to endure and thrive under stressful conditions). A core prediction of both SEH and MESH is that species are excluded towards more reflective conditions due to the harsher swash climate and coarser sediments^4^. This is expected to generate a source-sink dynamic that has been observed for some individual macrobenthic species^15,16^, but yet has to be investigated in a metacommunity framework in sandy beaches.

Global diversity patterns in sandy shores seem to validate sandy beach hypotheses and the role of beach morphodynamics as the main explanatory factor structuring intertidal macrofaunal assemblages: a consistent increase in species richness, abundance and biomass is observed from reflective to dissipative conditions^17,18^. Nevertheless, for such hypotheses to be valid, the following assumptions also need to be fulfilled:

1. Given that sandy beach fauna is tightly related to morphodynamic characteristics, environmental variables should be the main structuring factor of intertidal macrobenthic assemblages. Furthermore, at a regional scale, beaches with similar morphodynamic characteristics should harbor similar macrobenthic assemblages.
2. Considering that the harsher environmental conditions towards reflective beaches determine the progressive exclusion of species, the species pool of a reflective beach would be a subset of species from nearby dissipative beaches. In other words, at a regional scale, biological communities from reflective beaches would be nested in the species pool of dissipative beaches.
3. Finally, for a nestedness pattern to arise from beaches with different morphodynamic characteristics, it is necessary that dissipative beaches not only have higher species richness, but also a higher number of exclusive species than reflective or intermediary beaches.

Recently, a few studies have been investigating some of those assumptions, but a comprehensive evaluation of the main hypotheses on distribution patterns of sandy beach macrobenthic communities is yet to be conducted. The influence of environmental and spatial variables (i.e., factors that affect a local assemblage regardless of environmental conditions - typically related to distance between sampling sites and dispersal of species) on sandy beach assemblages has been examined^19,20^; however, to our knowledge, only one attempt was made to estimate nestedness patterns in sandy beaches^21^. Since the publication of this study, a significant advancement has been made in statistical procedures to estimate nestedness, which allows for more robust calculations of distribution patterns^22^.

Knowledge about nestedness patterns on sandy beaches is important not only to improve our understanding of their ecological functioning, but also to provide valuable information for biodiversity conservation and management strategies. Nestedness patterns inform us on how species are distributed across different habitats^23,24^, information that is crucial to identify areas that should be prioritized for protection^25,26^. A nested pattern in sandy beaches would imply that the set of species inhabiting reflective beaches is a subset of the set of species inhabiting dissipative beaches; therefore, dissipative beaches should be priority targets for biodiversity conservation. On the other hand, a non-nested pattern, especially with exclusive species occurring in dissipative and reflective beaches, would suggest that a combination of different types of sandy beaches should be preserved. This information is crucial for sandy beach ecosystems, which are often neglected in coastal conservation programs^16,27^.

The aim of this study is twofold: (1) to improve the understanding of macrobenthic distribution patterns on a regional scale by comprehensively testing sandy beach ecological hypotheses, and (2) to provide information that can be used in conservation and management of coastal habitats. Specifically, we investigated (i) the influence of environmental and spatial variables on the distribution of sandy beach macrobenthic assemblages, (ii) the dissimilarity of macrobenthic assemblages in a set of nearby sandy beaches with contrasting morphodynamic characteristics; (iii) their nestedness patterns and the occurrence of exclusive species.

## Results

### Environmental characterization and biological data

Sampled beaches/sectors showed a range of morphodynamic characteristics (Table 1). According to Beach Index (BI) values and beach width, Toque-Toque and Picinguaba were characterized as reflective; Barra do Sahy, Baleia, Flecheiras 1 and 2, Cidade 2 to 4 and Camaroeiro were characterized as intermediary; whereas Cidade 1, Fazenda 1 and 2 and Palmeiras showed dissipative features. There was very little variability in BI values between periods. Beach slope and mean diameter followed the trend shown by the beach index, with reflective beaches having coarser and dissipative beaches having finer grains. Beach width varied greatly, ranging from 25 m (Picinguaba) to 140 m (Baleia and Fazenda 2). Calcium carbonate content also showed a great variation, ranging from 0.35% (Picinguba) to 7.33% (Baleia). Organic matter content (%) was generally low across the studied beaches (0.13% at Sahy to 1.39% at Palmeiras beach).

**Table 1:** Environmental characterization of the studied sandy beaches.

	Beach Width (m)	Slope (1/S)	M.D.(ϕ)	Sorting (ϕ)	CaCO_3_ (%)	O.M (%)	BI
Sahy	50	0.064	2.29 ± 0.68	0.63 ± 0.22	2.38 ± 1.00	0.44 ± 0.27	2.13 ± 0.08
Baleia	140	0.023	2.65 ± 0.10	0.39 ± 0.01	7.33 ± 1.82	0.49 ± 0.08	2.5 ± 0.01
Palmeiras	80	0.021	3.42 ± 0.02	0.34 ± 0.01	3.39 ± 0.09	1.20 ± 0.27	2.62 ± 0.01
Flecheiras 1	80	0.035	2.69 ± 0.15	0.77 ± 0.12	2.49 ± 0.01	1.09 ± 0.08	2.32 ± 0.02
Flecheiras 2	55	0.047	3.09 ± 0.03	0.68 ± 0.06	3.89 ± 0.95	1.07 ± 0.05	2.24 ± 0.01
Cidade 1	115	0.02	2.89 ± 0.08	0.69 ± 0.02	4.33 ± 0.92	0.74 ± 0.01	2.59 ± 0.01
Cidade 2	35	0.04	1.43 ± 0.24	0.90 ± 0.09	1.47 ± 1.06	0.48 ± 0.36	2.08 ± 0.04
Cidade 3	60	0.018	2.25 ± 0.46	0.96 ± 0.37	2.88 ± 0.62	0.53 ± 0.06	2.55 ± 0.06
Cidade 4	50	0.032	2.32 ± 0.01	1.07 ± 0.10	2.32 ± 0.27	0.59 ± 0.17	2.32 ± 0.01
Camaroeiro	50	0.028	2.43 ± 0.07	0.95 ± 0.01	3.21 ± 0.97	0.43 ± 0.06	2.39 ± 0.01
Fazenda 1	100	0.016	3.21 ± 0.06	0.29 ± 0.04	4.08 ± 0.54	0.43 ± 0.16	2.72 ± 0.01
Fazenda 2	140	0.013	3.20 ± 0.08	0.29 ± 0.01	1.18 ± 0.02	0.48 ± 0.03	2.81 ± 0.01
Toque-Toque	40	0.105	1.14 ± 0.25	0.85 ± 0.10	4.33 ± 0.59	0.22 ± 0.13	1.61 ± 0.05
Picinguaba	25	0.1	0.94 ± 0.04	0.67 ± 0.03	0.35 ± 0.11	0.32 ± 0.09	1.59 ± 0.01

M.D. stands for grain mean diameter, O.M. for organic matter content, and BI for beach index. Sampling periods are pooled, as no significant changes were found among periods for each variable.

A total of 2834 individuals, comprising 112 macrobenthic species, were collected throughout the study. Mollusks contributed to 53.6% of the total number of species (42 species of gastropods and 18 species of bivalves), whereas polychaetes accounted for 35.7% (40 species), crustaceans for 8% (9 species), and sipunculans and ophiuroids accounted for 1.8% each (2 species each). Mollusks and polychaetes also accounted for more than 95% of macrobenthic abundance, with a slightly higher number of mollusks being sampled (1440 and 1297 individuals, respectively).

### Influence of spatial and environmental variables on sandy beach macrobenthic fauna

Beach width, slope and mean diameter were the best explanatory environmental variables of macrobenthic distribution. Selected eigenvectors (dbMEM) were mainly related to large-scale patterns (Table 2). The spatial component alone [c] had an overall higher contribution to the variation in species distribution (0.18) than the environmental component alone [a] (0.08). Both components had significant values (Table 3). The shared explained variance [b] was 0.11 and the unexplained variance [d] was 0.63.

**Table 2:** Variables selected by forward procedure for the environmental and spatial components.

	Cum. R²	F-Test	P-value
Environmental variables			
Mean Diameter	0.932	2.672	0.014
Beach Slope	0.187	2.883	0.012
Beach Width	0.282	3.191	0.004
Spatial eigenvectors			
dbMEM 1	0.111	3.257	0.002
dbMEM 2	0.198	2.706	0.003
dbMEM 3	0.283	2.831	0.005
dbMEM 6	0.344	2.183	0.007
dbMEM 4	0.395	1.889	0.03
dbMEM 22	0.446	1.911	0.033

Spatial eigenvectors (dbMEMs) with low numbers represent large-scale, whereas high numbers represent small-scale patterns.

**Table 3:** Variation partitioning and permutation analysis results for the explained variance of environmental and spatial components on the distribution of sandy beach macrobenthic community.

Component	Explained Variance	Df	F-Test	p-value
Environmental [a]	0.08	3	1.947	0.003
Spatial [c]	0.18	6	2.147	>0.001
Shared [b]	0.11			
Residual [d]	0.63			

### Community dissimilarity among beaches

**Figure 1:**
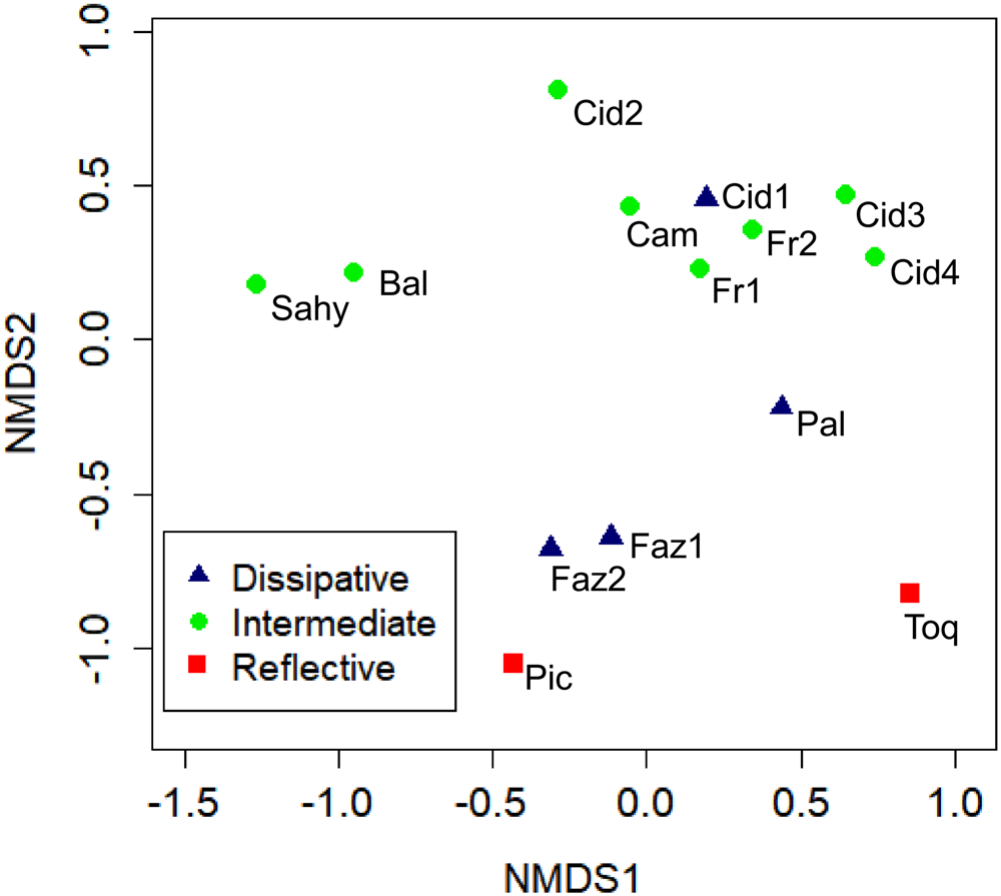
Dissimilarity in community composition among sampled beaches. NMDS stress = 0.109. Refective beaches: Toque-Toque (Toq) and Picinguaba (Pic); Intermediate beaches: Barra do Sahy (Sahy), Baleia (Bal), Flecheiras 1 and 2 (Fr1 and Fr2), Cidade 2 to 4 (Cid 2 to Cid4) and Camaroeiro (Cam); Dissipative beaches: Cidade 1 (Cid 1), Fazenda 1 and 2 (Faz1 and Faz2) and Palmeiras (Pal).

Dissimilarity in species composition of macrobenthic communities seemed to be more related to spatial distance than beach type. Macrobenthic assemblages were more similar among intermediate beaches, with the exception of Sahy and Baleia, the two intermediate beaches located further away from the others. Fazenda beach sectors (characterized as dissipative) had a stronger similarity with the neighboring beach, Picinguaba (reflective), than with the dissipative beach Palmeiras. Beach type, however, was an important factor, as Palmeiras beach was different from the neighboring intermediate beaches, and Toque-Toque, the only reflective beach on the southern part of the study area, showed a high dissimilarity with other beaches (Fig. 1).

### Nestedness patterns

NODF (Nestedness metric based on overlap and decreasing fill) values show a non-nested pattern of species distribution along the investigated environmental gradients of each variable. In fact, the observed values were consistently lower than the expected values, with negative z-scores, indicating a tendency towards an anti-nestedness pattern. The differences between NODF observed and expected values, however, were only marginally significant for all ordering variables in both periods (p < 0.10). The results for Matrix Temperature (T) were not significant, and a non-nested pattern was recorded for most periods and variables. With the composition from both periods pooled, the tendency towards anti-nestedness was much lower, and the observed nestedness was not different from the expected value under the null model for every variable and for both NODF and Matrix Temperature (Table 4).

**Table 4:** Nestedness metrics for each beach characteristic used as a gradient to rank sites to identify nestedness patterns.

	NODF				Temperature (T)			
	*Obs*	*Exp*	*Z-score*	*P(H0)*	*Obs*	*Exp*	*Z-score*	*P(H0)*
*Beach Index*								
*Fall*	20.41	20.87	−1.43	0.073	39.14	39.88	−0.54	0.296
*Spring*	24	24.67	−1.28	0.099	31.82	32.12	−0.16	0.437
*Total*	25.96	26.09	−0.41	0.339	36.79	37.66	−0.71	0.241
*Mean Diameter*								
*Fall*	20.38	20.82	−1.46	0.071	44.73	42.65	1.63	0.051
*Spring*	23.84	24.47	−1.3	0.096	32.24	34.33	−1.02	0.154
*Total*	25.96	29.06	−0.34	0.367	40.57	40.42	0.12	0.453
*Beach Slope*								
*Fall*	20.44	20.9	−1.53	0.063	37.17	38.57	−0.97	0.165
*Spring*	23.97	24.64	−1.38	0.083	33.15	32.38	0.41	0.34
*Total*	25.98	26.09	−0.37	0.356	36.29	36.22	0.06	0.476
*Beach Width*								
*Fall*	20.42	20.85	−1.43	0.076	42.92	42.85	0.04	0.482
*Spring*	23.84	24.39	−1.17	0.12	33.89	36.8	−1.52	0.064
*Total*	25.95	29.07	−0.38	0.352	39.73	41	−0.97	0.165

### Relationship between number of species and beach type

**Figure 2:**
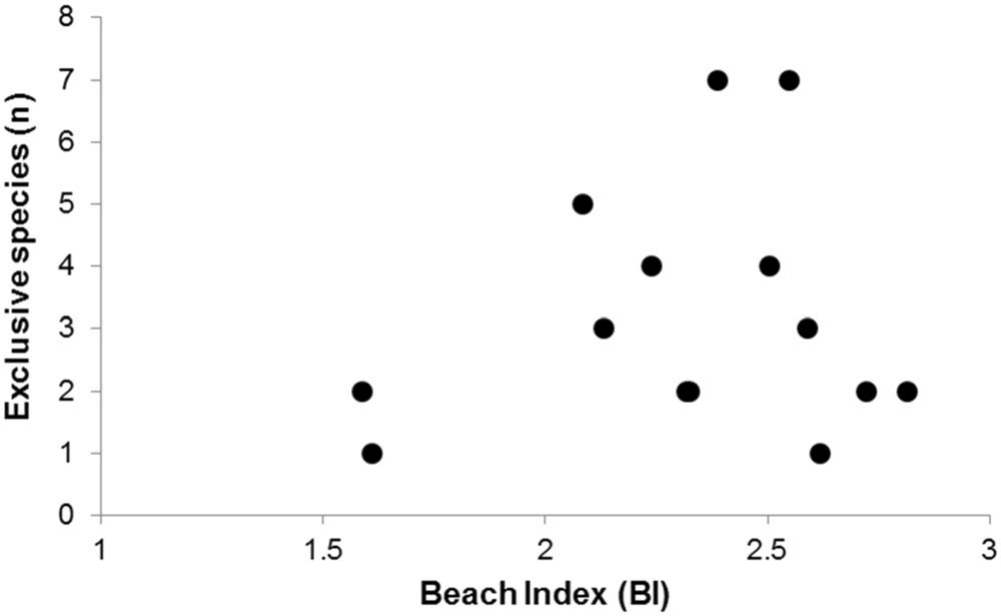
Number of exclusive species found at each beach, classified based on the beach index (BI). No statistical trend is observed in this relationship.

The number of species was positively correlated to BI (n = 28, r = 0.40, p = 0.037), negatively correlated to beach slope (n = 28, r = −0.46, p = 0.014), but not to grain size (n = 28, r = 0.30, p = 0.126) and intertidal width (n = 28, r = 0.11, p = 0.580). No increase of exclusive species towards dissipative beaches was observed. In fact, most beaches had 1 to 3 exclusive species registered, regardless of BI value (Fig. 2). Contrary to our predictions, the highest number of exclusive species was registered on intermediate beaches.

## Discussion

Significant changes in macrobenthic assemblages were associated with different sandy beach types, but morphodynamic characteristics alone did not explain the observed biological patterns. A higher number of species was found in dissipative beaches, and environmental characteristics significantly explained the structure of sandy beach assemblages, partially supporting sandy beach hypotheses. However, distance between beaches had an overall higher contribution to the variation in species distribution than environmental variables. Furthermore, we did not find a nested pattern in the morphodynamic continuum nor an increase of exclusive species towards dissipative beaches.

The number of macrobenthic species was significantly correlated with BI and beach slope, highlighting that environmental characteristics are an important factor structuring intertidal macrobenthic assemblages^4,7,28^. This importance was further reinforced by the significant contribution of the environmental variables alone in explaining the distribution of macrobenthic assemblages. Nevertheless, our results also revealed that the distribution of macrobenthic organisms in the study area was mainly related to spatial variables. Similar results, i.e. strong influence of spatial variables on marine soft sediment assemblages, have been reported in recent studies^19,20,29,30^ and are an indication that spatial processes are an important structuring factor in this system.

Several authors have pointed out that the variation accounted by spatial processes may arise from two main sources: it can be attributed to pure spatial processes related to dispersion of organisms or to some spatially structured unmeasured environmental variables^31–33^. Here, it is likely that both sources affected sandy beach macrobenthic assemblages.

Sandy beaches are thought to be systems highly connected by species dispersal, forming metapopulations that are limited by environmental conditions^5,16^. Although this process holds true at the population level for some macrobenthic taxa^15,34^, it may not occur at a community level. The stronger influence of the spatial component alone in comparison to the environmental component, and the high similarity of macrobenthic assemblages in neighbouring beaches, despite their morphodynamic state, suggest that local recruitment and small-scale dispersion are predominant processes for macrobenthic metacommunity dynamics at the study area. In this sense, our results agree with suggestions that sandy beaches are somewhat isolated systems^35^ (although the degree of isolation is likely to vary among species and dispersal strategies^20^). Our results also agree with evidence that macrobenthic species tend to recruit locally, despite dispersal potential, due to the favourable conditions of the parental area^36,37^. It is important to notice that intertidal species are more limited in dispersion capability than subtidal ones^38^, and studies evaluating the subtidal community may find spatial processes to contribute differently.

On the other hand, spatially structured unmeasured environmental variables could also be responsible for the stronger influence of the spatial component. Although we included many environmental variables that are commonly found to affect the species distribution of soft sediment macrobenthic assemblages (e.g., slope, width, BI and sediment), many others which could be spatially structured were not considered. Physical forces such as local currents, winds, and small-scale turbulent processes in the water column are known to affect recruitment, distribution and mortality of marine invertebrates^39–43^, and, therefore, may contribute significantly to their spatial patterns. Oxygen and nitrogen content in the sediment, salinity and algal biomass are other important environmental factors that were not considered here^17,19,44^. Thus, it is likely that the environmental control observed here is underestimated and it is important that future studies on macrobenthic assemblages include more variables than only morphodynamic characteristics.

Although the inclusion of a larger set of environmental variables may increase the importance of the environmental component explaining the patterns of richness and distribution of sandy beach macrobenthic assemblages, our results clearly show that spatial processes, which are often neglected in sandy beach ecological studies^4,7^, can exert a significant influence on these assemblages. Here we considered distance as the spatial component; nonetheless, the use of connectivity measures in the spatial structure has been shown as a promising tool in the evaluation of spatial processes in metacommunities^45,46^ and should be tested in further studies on sandy beaches.

Nestedness usually arises as a result of a species filtering process along an environmental gradient due to differences in the tolerance of species to that gradient^22,47^. Sandy beaches show a clear environmental variation along the morphodynamic continuum; however, our expectations of significant nestedness patterns on sandy beach macrobenthic fauna were not corroborated, which shows that macrobenthic assemblages of reflective beaches are not a subset of those from dissipative beaches. The higher contribution of spatial processes compared to environmental variables to the distribution of macrobenthic assemblages in the study area is likely to reduce the influence of the species filtering process, resulting in the non-nested pattern observed.

In some cases, the macrobenthic metacommunity presented a tendency towards anti-nestedness. This concept is used in different situations, and thus the origin of such pattern is hard to distinguish^48,49^. One explanation may lay in low prevalence, where species presented in a single site increase the tendency towards anti-nestedness^50^. Exclusive species were found on almost every beach and during every period in the study area. This pattern resulted in a sparsely filled species by site matrix (i.e. with many non-occurrences). When more than 40% of sites within a dataset are required to include all species, these datasets show a tendency towards anti-nestedness patterns^49^. These factors may be responsible for the observed tendency towards anti-nestedness patterns in the distribution of macrobenthic species. In this sense, anti-nestedness can be seen as a process of species replacement and high beta diversity^51^.

Besides increasing the ecological understanding of sandy beach assemblages, our results bring important implications for conservation programs and management plans of sandy beaches. When nestedness occurs, species-rich assemblages can be prioritized for conservation, as species-poor assemblages comprised only a subset of species^52^. Our results, however, show that different beach types are likely to contain complementary sets of species, as showed by the non-nested patterns and the dissimilarity among sites. This complementarity is further reinforced by the presence of exclusive species in almost every beach analyzed. Therefore, sandy beaches with different morphodynamic characteristics need to be protected in order to maintain overall biodiversity, and management programs with this goal should not be based only on sandy beach type.

Overall, we found partial support for hypotheses explaining the distribution patterns of intertidal macrofaunal assemblages. Morphodynamic characteristics were an important factor structuring the fauna, but spatial distance had a stronger influence on macrobenthic distribution. We found no support for the hypothesis of a nestedness pattern related to morphodynamic beach type. Instead, we detected a non-nested distribution, with exclusive species occurring on almost every beach. These results indicate that processes related to dispersal may be more important than local environmental features and that different sandy beaches harbor different assemblage composition. Therefore, we suggest that conservation practices across regional/large spatial scales should consider beaches with distinct morphodynamic features in order to preserve regional biodiversity. Future studies should investigate a more complete set of parameters attempting to find conservation targets that adequately protect sandy beach ecological diversity. This is an imperative task on which our quality of life in the long term relies^16^.

## Methods

### Study area

**Figure 3:**
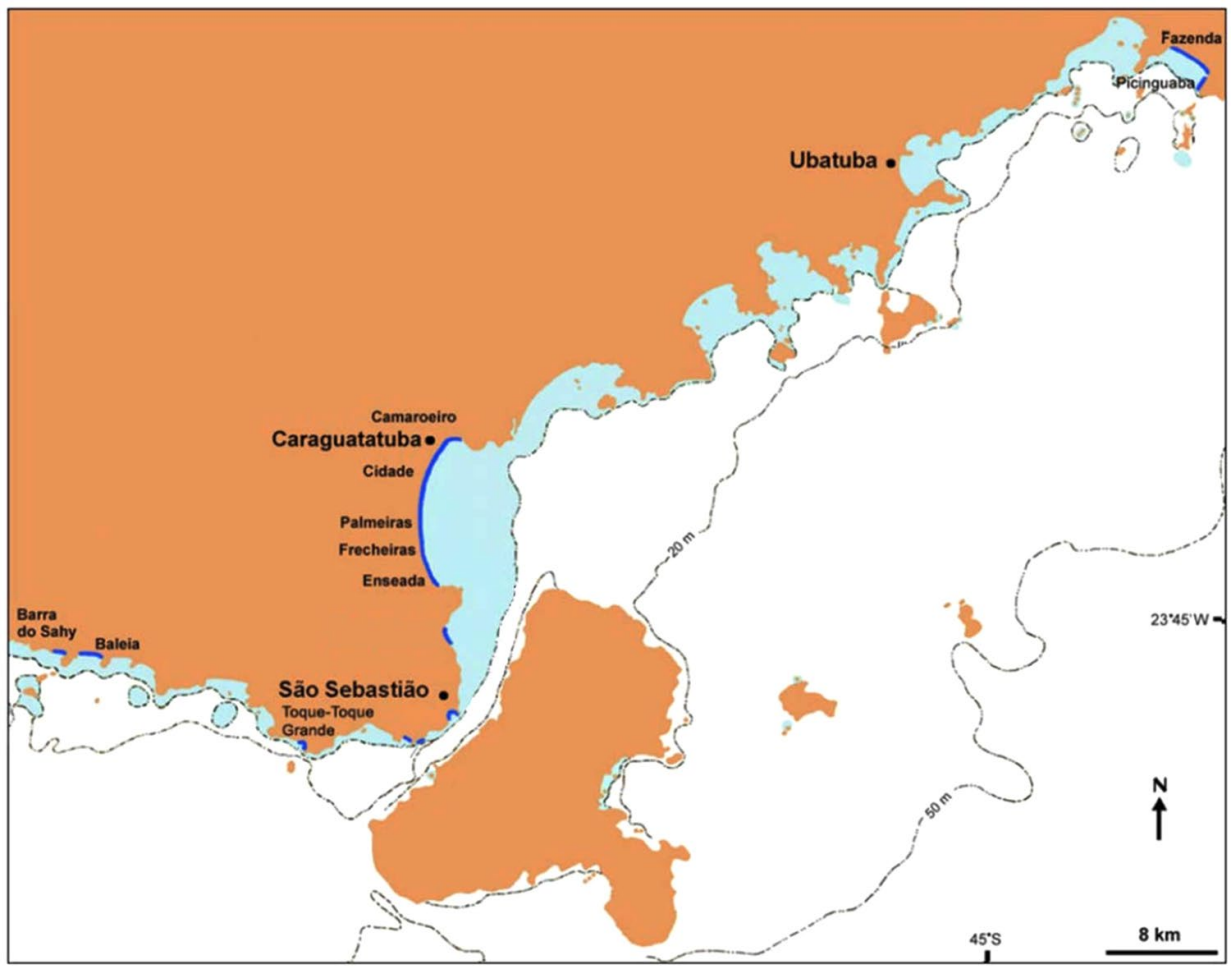
Location of the beaches sampled on the northeast coast of the State of São Paulo (SE Brazil), along the three municipalities (São Sebastião, Caraguatatuba and Ubatuba) (Modified from Amaral & Denadai^69^). Sampled beaches: Barra do Sahy (23°46′30″ S, 45°41′40″ W), Baleia (23°46′28″ S, 45°40′31″ W), Palmeiras (23°41′38″ S, 45°25′46″ W), Flecheiras 1 (23°38′37″ S, 45°25′23″ W), Flecheiras 2 (23°38′15″ S, 45°25′11″ W), Cidade 1 (23°37′24″ S, 45°24′21″ W), Cidade 2 (23°37′22″ S, 45°24′2″ W), Cidade 3 (23°37′3″ S, 45°23′57″ W), Cidade 4 (23°37′31″ S, 45°23′54″ W), Camaroeiro (23°37′40″ S, 45°23′49″ W), Fazenda 1 (23°21′31″ S, 45°51′22″ W), Fazenda 2 (23°22′1″ S, 44°50′20″ W), Toque-Toque (23°50′4″ S, 45°30′39″ W), Picinguaba (23°22′39″ S, 44°50′17″ W).

This study was conducted at the Northern coast of the state of São Paulo, Southeast Brazil, in the municipalities of Ubatuba, São Sebastião and Caraguatatuba (Fig. 3). The area has a Cfa Köppen’s climate characterized as humid subtropical with hot summers and a lack of dry season^53^. Nine beaches in this area were selected for sampling (Barra do Sahy, Baleia, Palmeiras, Flecheiras, Cidade, Camaroeiro, Fazenda, Toque-Toque and Picinguaba). Beaches with a length greater than 1 Km (Cidade, Flecheiras and Fazenda) were divided in sectors due to different features registered along the shore, such as beach width and grain size, that could directly influence macrofauna abundance and occurrence^54,55^. Taking this into account, Cidade beach was divided in four sectors (Cidade 1 to 4), Flecheiras and Fazenda beaches in two (Flecheiras 1 and 2, Fazenda 1 and 2). All other beaches consist of 1 sector only, leading to a total of 14 sectors sampled. The position of each sector was recorded with a GPS (Garmin eTrex Legend, *datum* WGS84) and the same locations were sampled during each sampling event. Each beach sector was sampled twice, once during austral Fall/2001 and once during Spring/2001.

### Sampling desing

Sampling was done at three sampling sites (area of 100 m² each, 10 m on each side), equally spaced in the intertidal zone from the water line to the drift line, in the central area of each sector. Given the wide range of sizes of macrofaunal taxa, from a few millimeters to tens of centimeters, two complementary methods were used in order to obtain the most accurate estimate of biodiversity. Thus, in each sampling site, five samples were randomly collected with small corers (d = 10 cm, A = 0.008 m²) and sieved on mesh size of 0.5 mm to evaluate small-bodied fauna; and three samples were randomly collected with a large corer (d = 45 cm, A = 0.16 m²) and sieved on mesh size of 1.0 mm to evaluate larger species.

### Environmental characterization

To characterize the different sectors sampled, three sediment samples (A = 0.008 m²) were taken at each sampling site to evaluate granulometrical parameters (grain size and sorting coefficient), organic matter and calcium carbonate content (CaCO_3_). Beach type (dissipative, reflective or intermediate) was classified using the Beach Index (BI)^7^, according to equation (1):

1 $$BI={{log}}_{10}((Mz\,\ast \,TR)/S)$$

where *Mz* is the mean grain size (ϕ + 1); *TR* is the maximum spring tide (m) and *S* is the beach slope (1/tanβ). Higher values of BI denote more dissipative characteristics.

### Influence of spatial and environment variables on sandy fauna

To investigate the relative contribution of spatial distance and environmental variables on sandy beach macrofaunal assemblages, we used a variation partitioning procedure^56,57^ applied to the redundancy analysis (partial RDA)^31^. Abundance data from both periods were pooled to increase the number and abundance of species per site. Pooling was justified by Procrustes analysis results^58^, which showed that biological datasets from both periods were strong and significant correlated (Correlation = 0.823, P = 0.037). Sediment mean diameter was also not different between periods at each beach (Supplementary Table 1), given that beach width and slope were constant between periods, beach index (BI) was also not different. Other variables were not used in the redundancy analysis.

We used slope, beach width, beach index and mean grain size of each location as environmental variables. To derive spatial variables (proxy for spatial processes), we used distance-based Moran eigenvector maps (dbMEMs) for the matrix composed by the geographical coordinates of the sites. dbMEMs provide orthogonal vectors that maximize the spatial autocorrelation^57^. Large-scale spatial correlation is modelled by the initial dbMEMs, while the last dbMEMs correspond to fine-scale spatial correlation^57^. We used the longest distance connecting two neighboring sites as a threshold to truncate the distance matrix. Distances larger than the threshold value were replaced by an arbitrarily large value equal to four times the threshold and were disconnected in a neighbor matrix (i.e., truncated matrix)^59^. To minimize random effects by dominant taxa and make data more appropriate to be analyzed by linear ordination methods, community abundance data was transformed using Hellinger function^60^

The variation was partitioned in four fractions: (a) influence of environmental variables alone (slope, beach width, beach index and mean grain size); (b) shared influence of environment and spatial variables; (c) influence of spatial variables alone (distance-based Moran eigenvector maps, dbMEM); and (d) unexplained variation. The significance of each component was assessed by permutation tests (n = 10000) on the redundancy analysis model. Variable selection for both components was done using forward procedure with double stopping criteria, which considers Akaike values and Adjusted R² to select variables, reducing type I error likelihood^61^. Only variables selected by this procedure were included in the variation partitioning analysis.

### Community dissimilarity among beaches

We used a non-metric dimensional scaling ordination (NMDS) to assess the similarity in community composition among sectors. Abundance data for the full suite of species was log-transformed (log x + 1) to down weigh the influence of extreme values. Bray-Curtis index was used to calculate the community composition dissimilarity among areas. Similar to the redundancy analysis, data was also pooled for this analysis. The similarity in community composition and variation partitioning were carried out using the R Software v. 3.3.1^62^, with the additional packages *vegan*^63^ and *spacemakeR*^64^.

### **Nestedness analysis**

The community matrix of each sector was arranged with presence/absence records for each species. In order to evaluate nestedness, the species-by-site matrix needs to be ranked. The common procedure is to rank species in decreasing order of frequency and sites in decreasing order of richness, but ranking can be made according to a gradient of interest, in order to check whether nestedness arises from changes in the environment^22^. Thus, to test our hypothesis that reflective beaches have a subset of species from dissipative beaches, we ranked sites according to four proxies of beach morphodyamics: (1) decreasing value of Beach Index (BI); (2) decreasing value of mean grain size (ϕ); (3) increasing value of beach slope (1/S); (4) decreasing beach width (m). This procedure was carried out for individual as well as for pooled samples, in order to account for possible temporal variations in nestedness.

Two different metrics were calculated to estimate nestedness: the matrix temperature (T)^65^ and the nestedness overlap and decreasing fill (NODF)^23^. Each metric has a different theoretical basis to estimate nestedness and may show contrasting behavior^23,66^. *T* is a metric of matrix disorder, accounting for unexpected presences and absences from a perfectly nested matrix, ranging from 0° (perfectly nested) to 100° (minimum nestedness)^65^. NODF is based on the overlapped presence and the marginal totals between pairs of rows and columns, ranging from 0 (no nestedness) to 100 (maximum nestedness)^23^. The use of more than one metric to quantify nestedness is a common practice^49,67^, and we used the two metrics to check if an observed pattern is consistent among metrics. Simulations were made under the FF null model (Fixed-Fixed, n = 1000 randomizations), which randomizes preserving row and column total value. This null model has a more conservative approach (i.e., less prone to type I error) and also has a low sensitivity to matrix fill, size and shape^22,23^. Z-scores were calculated as a mean of standardization of values, following equation (2):

2 $$Z=(X-\mu )/\sigma $$

where *X* is the observed index value, µ is the mean and σ is the standard deviation of simulated expected index values. For NODF, positive values indicate a tendency towards nestedness, whereas negative values indicate a tendency towards anti-nestedness. For T, the opposite trend is expected. Nestedness metrics and null model analysis were carried out using the *NODF* software^68^.

### Relationship between number of species and beach type

The relationship between the number of species and the number of exclusive species with the Beach Index was carried out using Pearson linear correlation. Periods were pooled for this analysis to give a more accurate estimate of macrobenthic richness.

### Data availability

The datasets generated and analyzed during the current study are available upon request to the corresponding author.

## Electronic supplementary material

Supplementary Table
