# Supplementary material for: Nestedness patterns and the role of morphodynamics and spatial distance on sandy beach fauna: ecological hypotheses and conservation strategies
Source: Sci Rep. 2018 Feb 28;8:3759. doi: 10.1038/s41598-018-22158-3 (PMC5830602; doi:10.1038/s41598-018-22158-3)
Supplement: Supplementary file 1 — Supplementary Table [file 41598_2018_22158_MOESM1_ESM.doc]

**Nestedness patterns and the role of morphodynamics and spatial distance on sandy beach fauna: ecological hypotheses and conservation strategies**

Helio H. Checon, Guilherme N. Corte, Yasmina M.L. Shah Esmaeili, A. Cecília Z. Amaral.

**Supplementary Table.** Results for analysis of variance (d.f. = 1, residual d.f = 7) for differences of sediment mean diameter (Ф) between periods per beach zone (Upper, Mid and Lower Intertidal).

|  | **Fall** | | | **Spring** | | |  |  |
| --- | --- | --- | --- | --- | --- | --- | --- | --- |
| **Beach** | **Supra** | **Inter** | **Sub** | **Supra** | **Inter** | **Sub** | **F-Test** | **p** |
| Baleia | 2.76 ± 0.1 | 2.74 ± 0.2 | 2.64 ± 0.1 | 2.64 ± 0.1 | 2.58 ± 0.2 | 2.53 ± 0.2 | 0.101 | 0.904 |
| Camaroeiro | 2.33 ± 0.8 | 2.46 ± 0.4 | 2.6 ± 0.2 | 2.64 ± 0.3 | 2.29 ± 0.2 | 2.2 ± 0.1 | 0.884 | 0.440 |
| Cidade 1 | 3.05 ± 0.1 | 2.83 ± 0.1 | 2.61 ± 0.3 | 2.4 ± 1.5 | 3.11 ± 0.3 | 3.35 ± 0.1 | 1.778 | 0.209 |
| Cidade 2 | 1.33 ± 0.1 | 1.33 ± 0.9 | 1.84 ± 1.4 | 1.28 ± 0.3 | 1.28 ± 0.9 | 1.26 ± 1.7 | 0.134 | 0.723 |
| Cidade 3 | 1.63 ± 0.2 | 2.23 ± 0.5 | 1.93 ± 0.2 | 2.52 ± 0.6 | 2.59 ± 0.2 | 2.63 ± 0.3 | 0.802 | 0.471 |
| Cidade 4 | 2.49 ± 0.1 | 2.29 ± 0.2 | 2.21 ± 0.3 | 1.5 ± 1.0 | 2.62 ± 0.2 | 2.01 ± 0.5 | 2.471 | 0.126 |
| Fazenda 1 | 3.11 ± 0.1 | 3.1 ± 0.1 | 3.28 ± 0.1 | 3.23 ± 0.1 | 3.24 ± 0.1 | 3.3 ± 0.1 | 1.564 | 0.249 |
| Fazenda 2 | 3.29 ± 0.1 | 3.28 ± 0.1 | 3.21 ± 0.1 | 3.27 ± 0.1 | 3.29 ± 0.2 | 2.89 ± 0.7 | 0.688 | 0.521 |
| Frecheiras 1 | 1.98 ± 0.4 | 3.18 ± 0.2 | 3.23 ± 0.1 | 1.75 ± 0.2 | 2.44 ± 0.7 | 3.59 ± 0.1 | 2.073 | 0.168 |
| Frecheiras 2 | 2.44 ± 0.3 | 3.2 ± 0.5 | 3.56 ± 0.1 | 2.66 ± 0.4 | 3.12 ± 0.1 | 3.54 ± 0.1 | 0.347 | 0.713 |
| Palmeiras | 3.37 ± 0.1 | 3.45 ± 0.1 | 3.49 ± 0.1 | 3.35 ± 0.1 | 3.41 ± 0.1 | 3.47 ± 0.1 | 0.111 | 0.896 |
| Picinguaba | 1.13 ± 0.3 | 1.06 ± 0.4 | 0.73 ± 0.2 | 1.42 ± 0.2 | 0.69 ± 0.4 | 0.63 ± 0.2 | 1.679 | 0.227 |
| Sahy | 1.52 ± 0.1 | 2.87 ± 0.1 | 2.73 ± 0.2 | 1.44 ± 0.1 | 2.75 ± 0.1 | 2.48 ± 0.2 | 0.807 | 0.468 |
| Toque-Toque |  | 0.69 ± 0.1 | 1.24 ± 0.4 |  | 1.45 ± 0.1 | 1.18 ± 0.4 | 3.693 | 0.096 |
